# Monthly Summary

**Published:** 1887-06

**Authors:** 


### Monthly Summary.

Elements.—Physicists have for some time looked doubtingly on the elementary constitution of many of the sixty-five or more substances which are given in text-books on chemistry as elements; and the latest investigations of Mr. Crookes have served to make their right to be named elements still more questionable. To rightly understand the value of his conclusions, we must bear in mind that every metal in its spectrum, has written its autograph by its molecules. The bands of colour are ever there, and in the same position, though sometimes a single band has been separated by finer instruments into two bands, as in the well-known D line, which demonstrates the presence of Sodium. The problem attacked by Mr. Crookes was the metal Yttrium, which has a spectrum, made up of five lines, one of which is double. Using a very dilute solution of Yttrium and an equally dilute solution of ammonia, so that many hours elapsed before even turbidity was produced, after many days a precipitate resulted; this he distinguished as G*a*. The remaining clear fluid was then treated with a stronger solution of ammonia, and after a considerable time a precipitate, G*b*, was formed. This process was continued with a stronger and stronger ammonia precipitant until G*c*, G*d*, and G*e* were separately formed, and no further effect could be produced. Each of these precipitates was examined by the spectrum produced with the electric spark, and was found undeniably to correspond to one of the five lines, though not in the order in which they stand in the Yttrium spectrum. Had these precipitates been Yttrium in different conditions, there would have resulted the five-lined spectrum of Yttrium in each case, but as there only resulted one of the five in each case, it is fair to assume that these five substances are the components of the so-called Yttrium. If this assumption be a true one, we may have to unlearn our chemistry and go back to that of those grand old fellows, the

Alchemists, who held that gold only differed from baser metals by containing more of that subtle principle for which they were ever seeking, which Mr. Crookes has named protyle, and which he considers may have been the origin of all matter.—*Dental Record.*

---

ARTIFICIAL TEETH—*To Patients.*—The question first to be settled is, Who is to judge what is natural? Certainly not the patient, who has been without teeth for a long time, nor members of the family, nor intimate acquaintance, who have become accustomed to their appearance without these important organs.

It is a principle well established by experience, that a deformity, even hideous in its character, will, by daily intercourse, cease to attract notice. Take, for example, cross-eyed children; how seldom do parents notice this deformity so forbidding to strangers.

What consternation pervades the family circle when one of its members returns from the dental office having had his teeth extracted preparatory to an artificial set, while even yet the cheeks and lips are partly kept in place by the alveolar processes or sockets. A few weeks will suffice to reconcile the family to the disfigured countenance; and as the alveolar processes gradually absorb, and lips and cheeks shrink, the family as gradually come to look on the face as natural.

The patient again visits the dental office to have the artificial teeth inserted. Who now is best able to judge how the teeth should be arranged to restore the harmony of the features, the patient and accompanying friends, or the dentist, who, perhaps has not seen the face since the day the teeth were extracted? *We answer, the dentist.*

By dentist we do not mean every man who sticks two boards out against a building, in the form of a triangle, on which is gilded the word "*Dentist*"—but one who is a dentist in fact, and has studied the harmony of features; who, having the cheek-bones, nose and chin as guide marks, can fill out the mouth to harmonize with them; who, having observed the complexion of the skin, the color of the eyes and hair, and the age of the patient, can select teeth of such size, shape, and color, as he finds nature gives in similar circumstances.

The judgment of the dentist should be respected, and then, after sufficient time has elapsed to wear away impressions created by the absence of teeth, and an acquaintance has been formed with the altered expression, if the friends and acquaintances do not become reconciled to the denture, the dentist is responsible. On the other hand, should the patients presume to dictate how the teeth should be arranged, what should be their color, shape, and size; in other hands, should they presume to occupy the position both of patient and of dentist, producing, as it certainly would, an unsatisfactory result, the responsibility would be their own, and any after change would demand a corresponding fee.

However great the anxiety of the patient may be as to the final result, it cannot exceed that of the dentist, for on its successful issue depend his interest and reputation. Our advice then is, secure the services of a competent dentist, and then implicitly rely on his judgment.—*Allport's Dental Journal.*

---

LABORATORY HINTS.—It is just as important for the palatine surface of a plate to be smooth and polished, coming in such intimate contact, as it does, with the delicate mucous membrane, as the other surface. I believe, with many others, that with perfect cleanliness you rarely meet with a sore or scalded mouth, and with a rough, uneven surface, it is impossible to have perfect cleanliness. I have tried many ways to accomplish this object, but have found the following method the best and simplest, all things considered:

Always add the plaster, (a little at a time, stirring constantly,) to the water, not the water to the plaster. After getting the impression, (always taken in plaster,) give it a coat of shellac varnish, not too thin, and while it is drying run a ring of sheet wax around it, an inch or so deep. After the varnish is perfectly dry, instead of oiling, sprinkle powdered French chalk freely over the impression, then with a soft brush—I use a badger's hair shaving brush—rub every part of the impression thoroughly, finally shaking out the surplus; then mix the plaster pretty thin, and pour, tapping the cup gently until the plaster commences to set. A ter it has hardened and you have cut away the impression, you will find the cast to have a perfectly glazed surface, free from air bubbles. Now proceed as usual and when the wax is removed dust the cast with chalk, the same as you did the impression. You will find after the plate is vulcanized, all that will be necessary to have a beautifully polished palatine surface, will be a short use of the brush wheel.

A word as to broken blocks. I am positive the only cause that ever broke a block is pressure--either too much rubber was used, the flask closed too suddenly, or at too low a temperature; or, lastly, the most frequent of all causes, neglect to remove all wax from the surface of the blocks. If any wax is left on the face of a block, there will be a space where the wax is melted out, and when the rubber is placed in behind it the block is almost sure to give way. Have the plaster set perfectly to the face of the teeth, and you will have no more broken blocks, at least that has been my experience. I saw in one of the journals, not long ago, where some one tried to explain that it was the "sudden pouring of hot water on the blocks that did the damage," citing as an example, that "his wife never poured hot water on glassware without expecting it to break." I will say to this gentleman, if he will watch his wife the next time she washes dishes he will find that she pours the hottest of water on porcelain with the greatest impunity, and if there are any glass teeth in the market I have failed to see them.—*C. C. E., in the Ohio Journal of Dental Science.*

---

STUDENTS' SOCIETY.—At the monthly meeting of the Dental Students' Society of the National Dental Hospital, Mr. James Rymer drew attention to a condition in the mouth of a boy aged 18, where there appeared an absence (supposed to developmental) of the front portion of the inferior maxilla, including the tip of the tongue. Mr. Alfred Prager read a paper on "Art in Relation to Dentistry," in which he urged his hearers to observe what great importance must be attached to the expression of the face by the arrangement of the dental arch, whether in regulation treatment or in the artificial substitution of the teeth. He cited as examples the various expressions obtained by the great masters by the disposition of the mouth and teeth, and pointed out that with the same features a change could easily be produced from one of the highest intelligence to that of idiocy, and from repose to anger.—*Dental Record.*

---

AMALGAM.—Dr. A. G. Bennett, in the "American System of Dentistry" says: "In spite of its ignoble nature, and in defiance of some prejudice and much opposition, amalgam has steadily won its way, till it is indorsed and used, to a limited extent at least, by many of the best operators, Though amalgam cannot be regarded as a specific for the arrest of caries, it is, with all its defects, the most durable plastic the inventive skill of the profession has produced. No condition or position of the teeth and cavities precludes its use, as it sets almost undisturbed by the presence of moisture; and on the score of comfort and economy to the patient nothing more could be desired. Its limitations, however, are often ignored. It may be said in general that amalgam, with all its real and alleged defects, including mercury as its essential ingredient, is to operative dentistry what vulcanite is to prosthetic dentistry. Both dispense with the highest skill, and lower and cheapen dentistry; but bring it within the reach of those who could not otherwise avail themselves of its service. It was opposed from the first, not because it failed to preserve teeth, but because when it preserved them best it discolored and disfigured them most; and it still often contains metals that have not been proven to be harmless, nor is it surprising that a material that saved teeth by destroying their beauty should have encountered decided opposition."

---

In using the elevating forceps in extracting under third molars, a piece of sheet tin or other substance should cover the back edge of the second molar to prevent its fracture.

